# Bleomycin-Induced Flagellate Dermatitis: Revisited

**DOI:** 10.7759/cureus.29221

**Published:** 2022-09-16

**Authors:** Pallavi Verma, Shalini Rajaram, Ayush Heda, Deepak Sundriyal, Parmita Tiwari, Ipshita Sahoo, Shilpa Panta, Mriganka Bordoloi, Himani Bhan, Dipendra Sharma

**Affiliations:** 1 Obstetrics and Gynaecology (Gynaecologic Oncology), All India Institute of Medical Sciences, Rishikesh, Rishikesh, IND; 2 Medical Oncology, All India Institute of Medical Sciences, Rishikesh, Rishikesh, IND; 3 Obstetrics and Gynaecology, All India Institute of Medical Sciences, Rishikesh, Rishikesh, IND

**Keywords:** whip-like pattern, hyperpigmented linear rash, bleomycin side effect, bleomycin rash, ovarian germ cell tumour, flagellate dermatitis, bleomycin

## Abstract

Flagellate dermatitis caused by bleomycin is a rare side effect with a distinctive pattern of whip-like, linear streaks. The clinical presentation has become uncommon nowadays as bleomycin use in conventional chemotherapy regimens has decreased. We present a case of a 30-year-old female diagnosed with ovarian germ cell tumour, managed with bleomycin, etoposide, and cisplatin (BEP) and later developed a widespread rash indicative of classic flagellate dermatitis. This brief report emphasizes the significance of detection and management of this transient dermatological complication in patients receiving bleomycin.

## Introduction

The cytotoxic effect of bleomycin is caused by the generation of activated oxygen free radicals that break single and double-stranded deoxyribonucleic acid (DNA), resulting in cell destruction. Hodgkin's lymphoma, germ cell tumours, and skin, head, and neck squamous cell carcinomas are treated with bleomycin. Due to low bleomycin hydrolase, a metabolizing enzyme, bleomycin-induced toxicity is more evident in the lungs and skin. Raynaud's phenomenon, nail bed changes, hyperkeratosis, skin peeling on palmar and plantar surfaces, digital gangrene, and pigmentary changes are all manifestations of bleomycin-induced dermatological toxicity. Flagellate erythema is an uncommon but distinct bleomycin toxicity, with an incidence of 8-20% documented in the literature [1]. Moulin et al. originally documented flagellate erythema as a side effect of bleomycin in 1970 [2]. However, this unusual reaction has become less common in clinical practice.

## Case presentation

A 30-year-old unmarried female presented with abdominal distension and pain after three months of laparotomy for pelvic mass with gross ascites at a local hospital. The histopathology report revealed high-grade immature teratoma of the ovary. On examination, she was cachexic with Eastern Cooperative Oncology Group (ECOG) performance status 3. Abdominal examination revealed tense ascites and an ill-defined fixed 11x10 cm pelvic mass. Tumour markers were substantially elevated as shown in Table 1.

**Table 1:** Pre-chemotherapy tumour markers AFP: alpha-fetoprotein; LDH: lactate dehydrogenase; β hCG: beta human chorionic gonadotropin; CEA: carcinoembryonic antigen; CA: cancer antigen

Tumour marker	Value	Reference range
AFP (ng/ml)	9872	0 - 8.1
LDH (U/L)	990	0 - 247
β hCG (mIU/mL)	21	0 - 5
CA 125 (U/ml)	13	0 - 30.2
CA 19.9 (IU/ml)	180	0 - 37
CEA (ng/ml)	3	0 - 5

**Figure 1:**
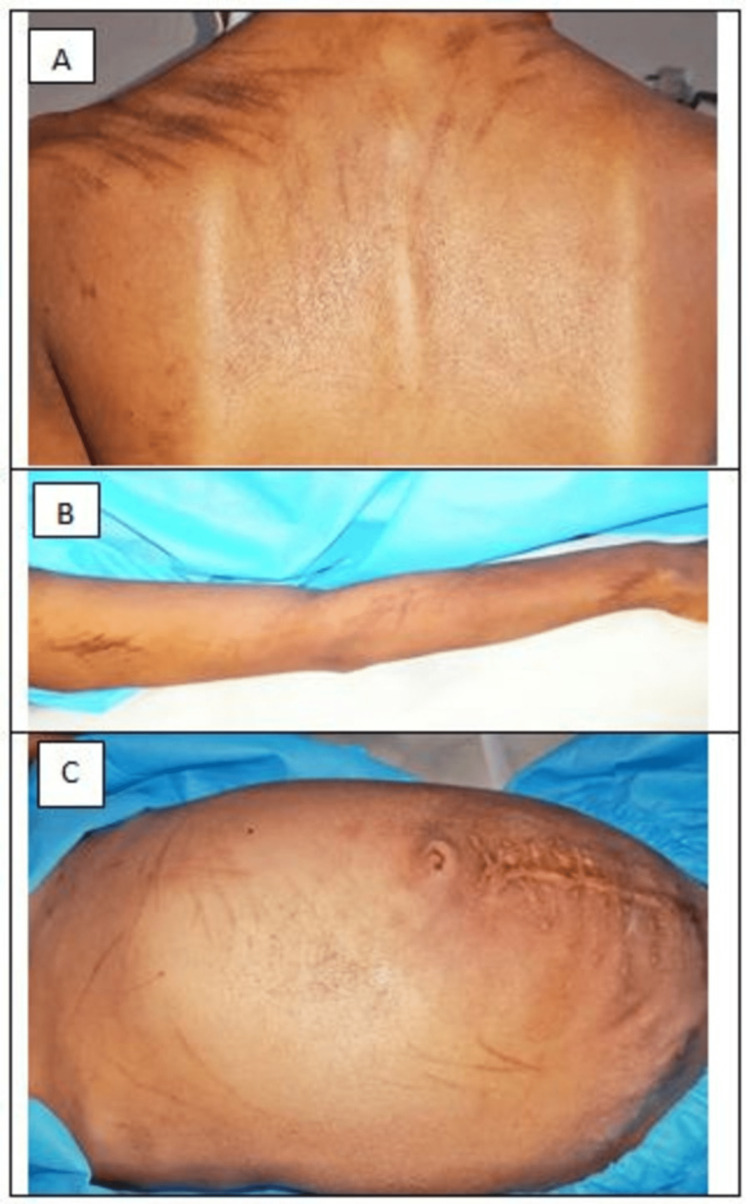
Multiple, well‑demarcated, hyperpigmented linear bleomycin-induced flagellate dermatitis on upper back (A), arm (B), abdomen (C)

**Figure 2:**
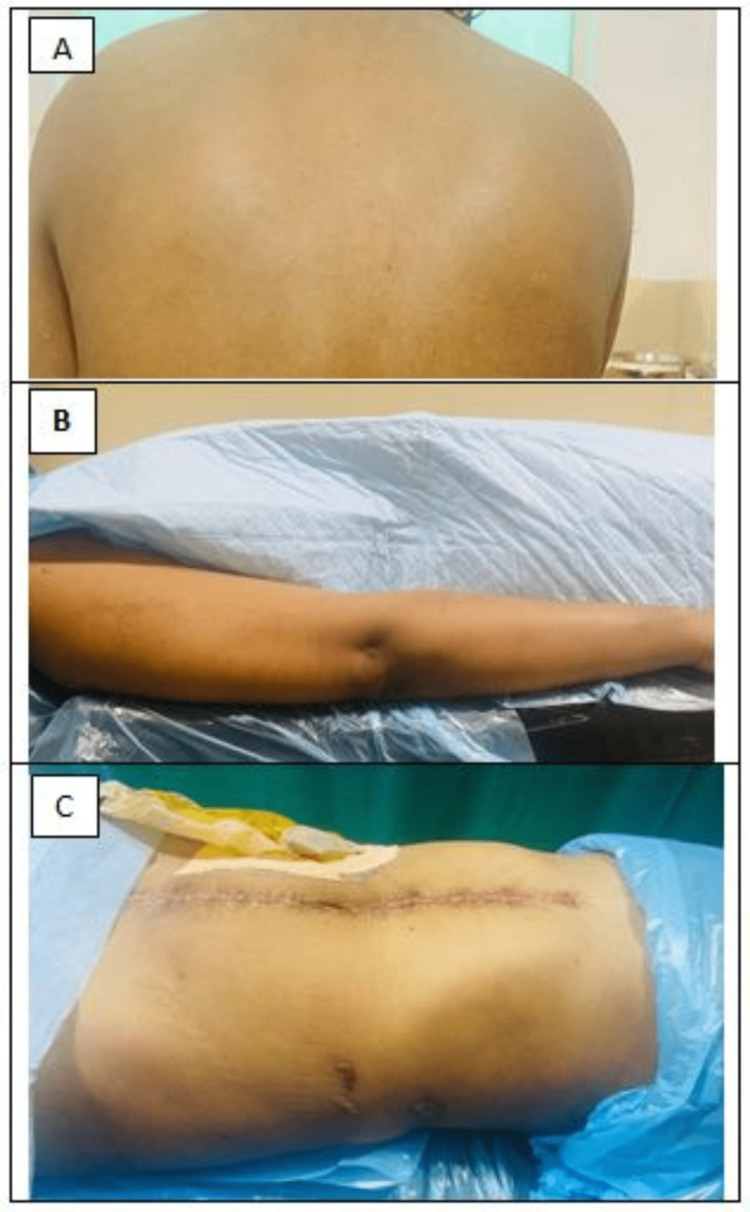
Disappearance of rash after six months from upper back (A), arm (B), and abdomen (C)

Contrast-enhanced computed tomography (CECT) scan of the thorax, abdomen, and pelvis showed a large heterogeneously enhancing pelvic mass 9x5.8x7.4 cm involving bilateral adnexa with multiple large peritoneal, intrahepatic, and splenic deposits, along with gross ascites and nodularity of omentum and peritoneum. Omental biopsy was reported as metastatic immature teratoma. The patient received two cycles of chemotherapy with a three-week BEP regime (Bleomycin 30 units IV weekly, Etoposide 100 mg/m2 IV day 1-5 and Cisplatin 20 mg/m2 IV day 1-5). She developed progressive, linear hyperpigmented streaks involving the upper back, arm, and abdomen (Figure 1) associated with itching after the second cycle of chemotherapy. The third cycle of chemotherapy was deferred as the patient had increasing abdominal distention, and imaging was suggestive of progressive disease; however, tumour markers decreased markedly. A repeat biopsy from the liver deposit and adnexal mass was reported as mature teratoma, and a diagnosis of growing teratoma syndrome was made. She was prescribed topical emollient cream for local application and oral tablet levocetirizine daily for a week for the pruritis. The lesions disappeared completely after stopping bleomycin therapy within six months (Figure 2). The patient underwent extensive cytoreductive surgery for the growing teratoma syndrome and is currently under follow-up and disease-free for six months.

## Discussion

Bleomycin is a *Streptomyces verticillus*-derived antibiotic. Inhibiting mitosis at low doses and limiting DNA thymidine uptake in the cell cycle S phase at higher doses results in anti-cancer effects. Bleomycin-induced flagellate erythema is a unique medication rash in which the patient's body appears as if it has been 'whipped'. Reported literature suggests that rows of neighbouring firm papules produce erythematous, linear, interlaced streaks. Although most cases appear to be heralded by an early onset of global pruritus, nonpruritic lesions have also been reported. Punctuate bleeding and pustules have also been reported [3]. There is no consistent pattern of lesions; they can be found on the face, the trunk, or the extremities. However, few studies have highlighted the presence of lesions over bony prominences. The presence of dermatographia is rare. It is debatable if scratching has any impact on linear pattern development. Affected areas become pigmented when the rash gets less erythematous. Hyperpigmented areas can last for up to six months. This side effect may be caused by localized melanogenesis, inflammatory pigmentary incontinence, alterations to normal pigmentation patterns, or cytotoxic effects of the drug itself leading to neutrophilic eccrine hidradenitis [4]. Bleomycin slows epidermal turnover, leading to extended interaction between keratinocytes and melanocytes, as per histological and ultrastructural observations.

Flagellate dermatitis caused by bleomycin is a dose-dependent reaction that often develops at total doses above 100U. Contrary to these findings, some patients may develop it even at low doses as in our case [5]. Flagellate dermatitis has also been linked to other chemotherapy drugs like docetaxel [6]. Shiitake mushroom consumption, adult-onset Still's disease, dermatomyositis, and infection with human immunodeficiency virus (HIV) are additional causes of flagellate dermatitis [1,7]. Heat exposure can cause bleomycin-induced skin rash recall even months after treatment has stopped; therefore, it should be avoided [1].

Bleomycin-induced flagellate dermatitis needs to be promptly treated with antihistaminics and topical and oral corticosteroids [8]. In patients with a severe rash, short-term oral corticosteroid therapy with prednisolone (40-60 mg daily) has been tried [7,8]. Severe rash mandates avoidance of bleomycin [9]. Recurrence of rash has been reported with re-exposure to bleomycin and should be avoided [1,8]. A common aftereffect is permanent post-inflammatory hyperpigmentation in the affected area [1,3].

## Conclusions

Bleomycin-induced flagellate dermatitis is a rare side effect. The case images emphasize the significance of its clinical awareness, detection, and timely management in patients on chemotherapy with bleomycin. Treatment options range from topical emollients/ corticosteroids to antihistaminics and systemic corticosteroid therapy based on symptom severity. Clinicians must be aware of this unique adverse reaction associated with bleomycin so that it can be promptly discontinued if required and patients can be counselled about its reversible nature to allay their apprehension.
